# Can We Improve Vaccine Efficacy by Targeting T and B Cell Repertoire Convergence?

**DOI:** 10.3389/fimmu.2019.00110

**Published:** 2019-02-13

**Authors:** Katja Fink

**Affiliations:** Singapore Immunology Network, Agency for Science, Technology and Research, Singapore, Singapore

**Keywords:** B cell receptor (BCR), T cell receptor (TCR), immune repertoire analysis, personalized vaccination, infectious diseases

## Abstract

Traditional vaccine development builds on the assumption that healthy individuals have virtually unlimited antigen recognition repertoires of receptors in B cells and T cells [the B cell receptor (BCR) and TCR respectively]. However, there are indications that there are “holes” in the breadth of repertoire diversity, where no or few B or T cell are able to bind to a given antigen. Repertoire diversity may in these cases be a limiting factor for vaccine efficacy. Assuming that it is possible to predict which B and T cell receptors will respond to a given immunogen, vaccine strategies could be optimized and personalized. In addition, vaccine testing could be simplified if we could predict responses through sequencing BCR and TCRs. Bulk sequencing has shown putatively specific converging sequences after infection or vaccination. However, only single cell technologies have made it possible to capture the sequence of both heavy and light chains of a BCR or the alpha and beta chains the TCR. This has enabled the cloning of receptors and the functional validation of a predicted specificity. This review summarizes recent evidence of converging sequences in infectious diseases. Current and potential future applications of single cell technology in immune repertoire analysis are then discussed. Finally, possible short- and long- term implications for vaccine research are highlighted.

## Introduction

**Figure 1:**
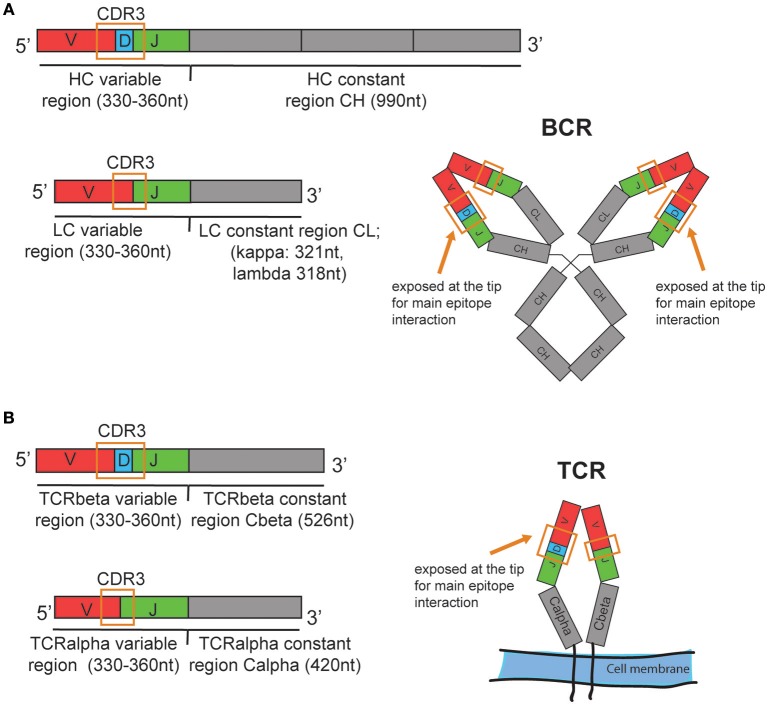
Schematic illustration of the human **(A)** BCR and **(B)** TCR mRNA organization and approximate transcript length. Nucleotide values are estimates based on the amino acid lengths reported in: https://www.uniprot.org/uniprot. Ab heavy chain (HC) variable region: 110–120amino acids (aa). HC constant region for IgG1 isotype: 330aa, Ab light chain (LC) variable region: 110–120aa, LC constant region kappa: 107aa, lambda 106aa. TCRbeta and TCRalpha variable region: 110–120aa. TCRbeta constant region: 176aa, TCRalpha constant region: 140aa. The mRNA is assembled from one allele of the variable (V), diversity (D), and joining (J) genes, which are illustrated in red, blue, and green, respectively. CDR3, Complementarity-determining region 3. Schematic structures of the expressed BCR and TCR are shown on the right with the CDR3 spanning V(D)J sequence. In the real structures, the CDR3 loops are exposed at the tips of the BCR or TCR.

The B cell immune repertoire can be analyzed quantitatively and qualitatively by sequencing the re-arranged variable region of the heavy chain (HC) of the B cell receptor (BCR) that is unique for each individual B cell or clonally expanded B cell (Figure 1). A B cell clone includes all B cells originating from one parent B cell that share the same heavy and light chain variable region nucleotide sequences. A clone can also include B cells that have a similar but not identical BCRs due to somatic hypermutation. The same variable (V) and joining (J) gene, the same length and a high similarity in the heavy chain CDR3 nucleotide sequence are generally used as criteria to define clonal relationship (1). Often, 85% amino acid sequence similarity is used as a simple inclusion criteria (2). In addition, computationally defined thresholds for clonal relationship for high throughput B cell repertoire sequencing data sets have been proposed (3). Since the same BCR heavy chain can be paired with different light chains (LC), sequencing of the light chain can confirm clonality, based on a unique HC-LC-chain pair per clone, and can provide more detailed information when assessing the breadth of an immune repertoire. Similarly, the T cell repertoire can be assessed by sequencing the beta chain of the T cell receptor (TCR). The alpha chain, although often not in contact with the antigen and therefore considered less informative (4), can confirm clonality and provide more detailed repertoire information.

Human repertoires are mostly assessed in blood, and depending on the sample volume the “repertoire” is in fact only a small part of the totality of all existing BCRs or TCRs in an individual. Nevertheless, bulk sequencing of receptors from cell populations have been very informative in capturing expanded clones in the context of disease. This has been most evident in the clinical diagnosis of B or T cell cancers. The transformed immune cells expand exponentially and the presence of a large malignant clone can easily be identified by TCR or BCR sequencing and the cancer type can be confirmed. The identified cancer cell's BCR or TCR sequence can later be tracked with high sensitivity during treatment and during remission, in order to assess whether the tumor is controlled (5, 6).

More recently, the identification and characterization of cancer neoantigen-specific T cells has highlighted the potential of TCR sequencing for the identification of tumor neoantigen-specific clones for cell therapy. Furthermore, sequencing can be used to track the expansion of the neo-antigen specific clones and thereby the success of cancer treatment. Independently, TCR repertoire information could enable tailored immunization strategies with neoantigens to match the T cell repertoire of a patient (6–8).

Although lymphocyte repertoire sequencing for cancer diagnostics and immunotherapy has become widespread, its use for diagnosis and prognosis of infectious diseases has not been established yet (9). Immune repertoire sequencing could help to identify the pathogen as demonstrated previously for CMV (10). In addition, it may further the understanding of the disease itself and enable prediction of the protective capacity of the immune response in the context of the previous infections. For example, influenza vaccine responses are affected by previous exposure (11). Re-activation of pre-existing immunity can boost a response, but it can also have a dampening effect due to antigen-masking by pre-existing or rapidly produced antibodies (12, 13). In the case of dengue infection where pre-existing immunity is a risk factor for subsequent infection, repertoire information could potentially distinguish patients with limited previous exposure from those with repeated exposure based on the mutation rate of the pathogen-specific BCRs. The latter patient group would likely have a low risk for severe disease, which can be a clinically useful information. Measurable parameters to assess pathogen-specific B cell and T cell repertoires can include the presence of converging sequences, repertoire breadth, and clonality.

Practical application of these concepts has been limited by a lack of methods and tools to identify pathogen-specific BCRs or TCRs in each person's unique repertoire. However, evidence of converging, pathogen-specific sequences, defined as sequence motifs that are observed in several if not all individuals that have experienced the same vaccination or infection, is accumulating.

The aim of this review is to provide an overview of the current evidence of converging sequences in infectious diseases, and to discuss potential applications and limitations of immune repertoire sequencing in vaccine development. The impact of single cell technology in the context is highlighted.

## Evidence of Converging B Cell Sequences as a Basis for Clinical Application of Repertoire Sequencing to Monitor Vaccine Efficacy

Excellent examples of how immune repertoire sequencing can inform immune function have been published in the context of broadly neutralizing antibodies (bnAbs) that are generated years after HIV infection (14, 15). Two repertoire-based studies published in 2011 provided the first evidence for the existence of a converging mode of antibody-virus binding (14, 15) (Table 1). The authors showed that bnAbs isolated from unrelated HIV-infected individuals bound to the CD4 binding site of the virus in a very similar way. In one study, Wu et al. found evidence of similar modes of binding between these bnAbs, although only around 50% of the amino acid sequence of the antibody heavy chain variable region (IgHV) were shared. Moreover, although one specific IgHV gene allele, VH1-2^*^2, was highly enriched amongst isolated bnAbs of the same binding class, high somatic mutation levels led to less than expected convergence in the amino acid sequence. Nevertheless, the authors managed to successfully predict HIV-binding monoclonal Abs (mAbs) based on the similarity of IgHV sequences from patients' B cells with known bnAbs sequences (15). In the second study, Scheid et al. described structural convergence of antibodies interfering with the binding of HIV to CD4 and conservation of amino acids in key CDR positions between same-binding-class antibodies isolated from five different donors (14). The study confirmed a preferential usage of germline gene VH1-2 and the closely related VH1-46 in CD4 binding site-specific antibodies. These and later studies provided evidence of convergence at least at the structural level, and demonstrated that it is possible to predict binding specificity from sequence patterns. However, given the relatively low sequence convergence, it was not clear how useful repertoire sequencing would be in general to predict Ab binding sites.

**Table 1:** Converging specific human BCR sequences from bulk cell analysis.

**Antigen**	**B cell enrichment (frequency)**	**Validation (single cell level underlined)**	**Number of donors**	**References**
HIV	Sorting with Resurfaced gp120 (RSC3) (0.13 and 0.15%)	Rec. mAb binding (a) from single B cells and (b) from bulk sequences, complementation with known bnAb VH/VL chains	2	Wu et al. (15)
HIV	Sorting with gp120 2CC and gp140 trimer	Rec. mAb binding; from single B cells	5	Scheid et al. (14)
HIV	Sorting with RSC3 (0.198%)	Rec. mAb binding (a) from single B cells and (b) from clonally related bulk-sequenced VH and VL sequences	1 (longitudinal samples)	Liao et al. (16)
HIV	None; MS of gp120 affinity purified plasma antibodies	Search plasma antibody sequences in single B cells, Rec. mAb binding	2	Sajadi et al. (17)
HIV	a) None; PBMC sequencing b) Sorting with gp120	Rec. mAb binding from single B cells	a) 6 for bulk sequencing b) 2 for single cell mAb validation)	Setliff et al. (18)
Dengue	None; PMBC for sequencing	Split patient samples into a training set and two test sets; no functional validation	44 patients, longitudinal samples	Parameswaran et al. (19)
Influenza	None; PBMC, plasmablasts for sequencing	Rec. mAb expression from single sorted plasmablasts	14 vaccinees, single plasmablasts from 5	Jackson et al. (20)
Influenza (TIV)	IgM-neg. Memory B cells,	single cell cultures, IgG in supernatant for analysis; mAbs from selected memory B cells	3 (binding convergence; sequence convergence with a previously described mAb	McCarthy et al. (21)

*Rec. mAb, recombinantly expressed monoclonal antibody; MS, mass spectrometry; TIV, trivalent inactivated influenza vaccine*.

Several converging BCR sequences have been identified and validated by sequencing bulk cell populations and single cells (Table 1). Since specific sequences usually comprise very small fractions of the entire repertoire (<1%, see Table 1), enrichment strategies are often used. Besides enrichment, cells that clonally expand several days after immunization can be tracked. This approach is based on the assumption that antigen-specific, BCR-mediated activation is the trigger for the clonal expansion (20, 22).

A purely computational approach is to compare the repertoires of an immunized group with those of a non-immunized control group, and to identify sequences that are significantly enriched in the immunized group. This strategy was applied, for example, in the context of dengue infection, where Parameswaran et al. used training and test sets to identify and computationally validate converging sequences (19).

So far, the identification of converging sequences in bulk cell populations has often been based on the BCR heavy chain sequence only, without proof that the “specific” BCRs are in fact binding to the antigen. To confirm pathogen-specificity with biological assays, individual B cells that express the sequences of interest must be isolated as single cells so that the information of paired heavy and light chain is conserved (23).

If a potentially pathogen-specific sequence is highly expanded, even a small number of several hundred isolated single cells could be sufficient to find at least one of the specific sequences from which recombinant receptors can be cloned. However, if a specific sequence is rare, hundreds or thousands of single immune cells need to be sequenced to be able to recover potentially binding clones that were identified in the more inclusive bulk cell repertoire. 96-well or 384-well sorting approaches are labor-intensive and therefore costly. The establishment of droplet technologies now facilitates the sorting of thousands of cells, while preserving the information of both heavy and light chain sequences [reviewed in (23)]. This then allows the expression of recombinant antibodies or the transfection of T cells with a TCR of interest and the validation of hypothetical converging sequences. So far, only a few converging sequences that were identified in bulk repertoires have been validated *in vitro* with biologically relevant readouts [Tables 1, 2, notably (4, 14–16, 18, 20)].

**Table 2:** Converging specific human TCR sequences.

**Antigen**	**T cell enrichment**	**MHC restriction**	**Sequencing strategy**	**Validation**	**Number of donors**	**References**
Influenza-(M1-58)	pMHC-tetramer selection	HLA-A^*^0201	Single cells (TCRα and TCRβ)	Comparison to non-epitope selected repertoire	15	Dash et al. (24)
CMV (pp65-495)	pMHC-tetramer selection	HLA-A^*^0201			10	
EBV (BMLF1-280	pMHC-tetramer selection	HLA-A^*^0201			6	
EBV (BMLF1-280	pMHC-tetramer selection	HLA-A^*^0201	Bulk (TCRβ) and single cells (TCRα and TCRβ)	a) Training; test set validation b) validation on new antigen = *M. tuberculosis* c) *in vitro* re-stimulation with predicted peptide/CDR3 glycine mutagenesis scan d) *de novo* synthesis and binding validation of predicted TCR	8	Glanville et al. (4)
Influenza-(M1-58)	pMHC-tetramer selection	HLA-A^*^0201			13	
Influenza-(HA306)	pMHC-tetramer selection	HLA-DRB1^*^0401			6	
*M. tuberculosis*	Peptide stimulation of PBMCs, then sort of CD154^+^ or cytokine secreting cells	Various HLA-class II			22	
YF-17D	PBMC and sorted CD4^+^ or CD8^+^ T cells	None, HLA-A^*^02 for validation	Bulk (TCRβ)	Compare expanded clones with clones found as follows: a) *in vitro* restimulation, sorted IFN-gamma^+^ cells b) sort activated cells c) sort tetramer-positive cells	3 pairs of monocygous twins; one of those pairs for validation	Pogorelyy et al. (25)
CMV	Unsorted, PBMC	–	Bulk (TCRβ)	a) Compare CMV-associated sequences with published, validated CMV-reactive TCRβ sequences b) peptide re-stimulation with blood from one donor	666 (CMV^+^ and CMV^−^ individuals)	Emerson et al. (10); Pogorelyy et al. (26)

*CMV, Cytomegalo virus; EBV, Epstein Barr virus; YF-17D, Yellow Fever virus vaccine strain 17D; M. tuberculosis, Mycobacterium tuberculosis*.

## Evidence of Converging T Cell Sequences as a Basis for Clinical Application of Repertoire Sequencing to Monitor Vaccine Efficacy

Antibody titers are correlates of protection for many if not most marketed vaccines. Antibody titers can be measured without extensive sample preparation time and with minimal equipment, and have therefore probably been targeted preferentially by companion diagnostics. However, this does not mean that antibodies are sufficient for protection. The T cell response is essential and possibly understudied as a secondary correlate of protection. This is exemplified in dengue infection where a vaccine based on B cell-mediated protection alone has limitations (27, 28). Aside from the requirement of CD4^+^ T cells for generating and maintaining B cell responses (29, 30), cytotoxic T cells (CTLs) are essential for eliminating virus-infected cells that can remain reservoirs of virus production. Virus-vectored or DNA vaccines which target CTL immunity are being developed for chronic infections, where the virus hides in infected cells without inducing cytopathic effects and without being accessible to antibody-mediated neutralization (31). All these approaches would benefit from TCR-based companion diagnostics.

In contrast to the burgeoning data on B cell repertoires, there is limited work on pathogen-specific T cell sequences which have been validated at a molecular and single cell level. Table 2 summarizes evidence of pathogen-specific converging TCR sequences in humans. In the context of infectious diseases, converging TCR sequences have been identified in healthy donors exposed to cytomegalovirus (CMV), Epstein-Barr virus (EBV), and *Mycobacterium tuberculosis* (TB), and in healthy donors immunized with influenza and yellow fever virus YV-17D vaccines (4, 10, 24). These studies show that converging TCR sequences exist for viral and bacterial antigens. It can be argued that CMV, EBV, and TB are latent infections that stimulate the immune response repeatedly over years, facilitating the selection of optimal BCRs and TCRs. Similarly, most individuals are exposed repeatedly to influenza. YF-17D is therefore an important proof of principle that single infections can generate measurable converging sequences.

Assessment of expanded T cell clones, while analyzing whether they are naïve or memory cells, may be a useful strategy to focus on long-term efficient vaccine-induced clones (32). DeWitt et al. found that after immunization with YF-17D, about two thirds of the expanded T cell clones could be associated with YF-17D vaccination due to their sequence overlap (33). The same study also found that only 5–6% of the expanded CD8^+^CD38^+^HLA-DR^+^ activated T cell clones were present in the memory CD8^+^ T cell pool 3 months after immunization. Importantly, more highly expanded clones were more likely to be recovered in the memory compartment.

If a CDR3 is shared between most individuals with the same infection it is likely that the given CDR3 is a public sequence representing preferentially assembled V(D)J alleles that result in the same -converging- amino acid sequences, instead of being a truly antigen-selected sequence (26, 34, 35). A recent study by Pogorelyy et al. illustrated the difference between convergent recombination and convergent selection based on large existing TCRß-sequence databases from CMV- and Type 1 diabetes cohorts (26). Convergently selected TCRß CDR3 sequences were rare compared to convergently reassembled TCRß CDR3 sequences. Some of the identified convergently selected TCRß CDR3 sequences had previously been validated by functional tests, showing that the approach could be useful to identify biologically relevant TCR sequences (26). As an illustration of how small the frequency of convergently selected public specific sequences is, Emerson et al. found that 488 (about half) of the previously reported 917 confirmed CMV-binding sequences could be found in a cohort of 666 individuals, but only 9 out of those 488 sequences (1.35%) were CMV-associated when comparing CMV^+^ with CMV^−^ donors (10). This implies that the other CMV-binding shared sequences were convergently reassembled public sequences that are overrepresented in naïve repertoires. It can be speculated that CMV is highly immunogenic because it binds to convergently recombined TCRs. In general, identification of low frequency shared sequences is severely hampered by limited sequencing depth and by subsampling. The volume of blood that can feasibly be collected from a person only represents a fraction of the complete repertoire. Therefore, repetitive blood draws from the same individual yield only about a tenth of unique sequences that overlap between individual draws (36).

## Computational Approaches to Predict Immune Receptor Function Based on Sequence

The CDR3 region of the heavy chain (H3) comprises the main interphase between antibody and epitope for a majority of antibodies and this region is therefore a major focus of prediction algorithms (37). However, due to the high sequence and length variability, predictions of H3 structures are less accurate than those for the two other, more conserved, CDR loops. Validation studies have shown that the accuracy of the models improves with the availability of structure homologs (38). Krawczyk et al. recently developed a pipeline called structural annotation of antibodies (SAAB) algorithm, to map amino acid sequences on existing antibody structures (39). Previously, DeKosky et al. showed a difference in physicochemical features between antigen-experienced and naïve antibody sequences, without predicting binding specificity *per se* (40).

Even if the antibody structure can be predicted accurately, predicting the specificity poses an additional challenge. Specific antibody-antigen docking prediction tools such as SnugDock (41) and ClusPro (42) should have an improved accuracy compared to generic protein-protein interaction prediction tools. Providing some proof of principle, potential therapeutic antibodies against dengue virus (43) and Zika virus infection (44) are recent examples of computationally optimized molecules.

To improve the identification of pathogen-specific TCR clones, Glanville et al. developed an algorithm called GLIPH (grouping of lymphocyte interactions by paratope hotspots) to predict binding specificity of given CDR3 patterns, based on 52 previously published TCR-pMHC structures (4). Interestingly, the study found a pathogen-associated enrichment of short motifs in CDR3 sequences, and these motifs corresponded to short stretches of amino acid sequences that are in contact with the peptide presented on MHC-I.

The motif approach is interesting given that, ultimately, very few complete CDR3 sequences are found in more than one individual.

## Immune Repertoire Sequencing in Personalized Vaccination: Current Status of Monitoring and Future Ambitions

Personalized vaccination is largely known in the context of therapeutic cancer therapy. Since every tumor or class of tumors has a unique protein expression pattern, proteins or peptides corresponding to unique tumor antigens could be used as tailored immunogens to trigger the expansion and activation of tumor-specific T cells (6).

In the context of infectious diseases, neutralizing B cell epitopes and T cell epitopes that are important for protection are limited in number. The number of pathogen-specific protective epitopes is determined by the size and complexity of a given pathogen and the respective vaccine formulation. Pathogen mutation, which is a source of high diversification in viruses, can be controlled by inactivation of the pathogen.

In contrast to tumors where T cell tolerance and the absence of neoantigens can limit therapeutic vaccination, there are more options to modify the antigens of a pathogen and adapt a vaccine to optimally engage the immune repertoire of an individual, as proposed for new germline-targeting HIV vaccine (45) and influenza vaccine (46) approaches. The idea is to engage naïve B cells and to use a stepwise boosting protocol to mutate the B cell receptor toward a novel epitope for the antigen that cannot be recognized by germline BCRs.

**Figure 2:**
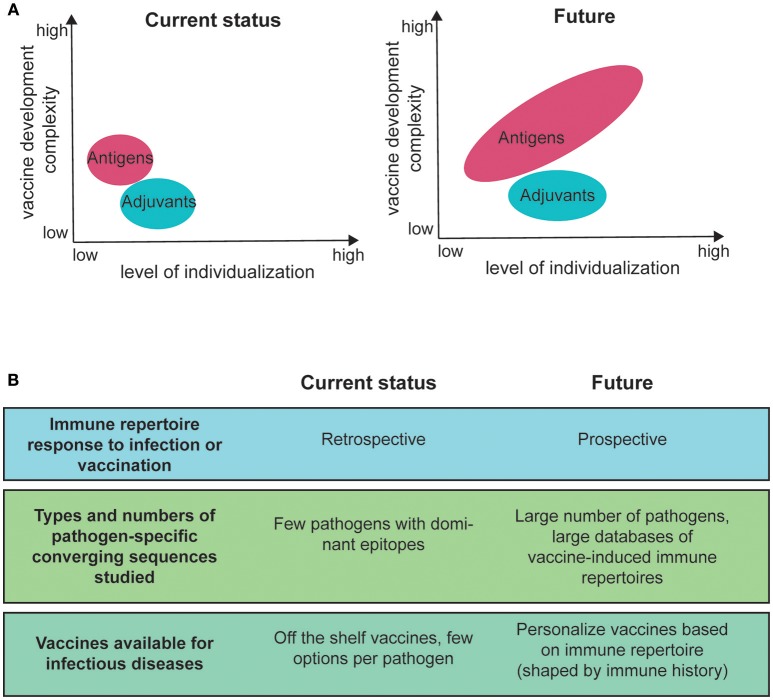
Current and future immune repertoire research areas for personalized vaccines for infectious diseases. **(A)** level of personalization compared with the level of complexity for vaccine development. The size of the antigen and adjuvant fields illustrates the number of different options. **(B)** areas of current research in the field of immune repertoire analysis for infectious diseases and potential areas for future application.

With the availability of multiple, immunologically diverse adjuvants, personalization of the adjuvant is an additional option (47) (Figure 2A). Adjuvant efficacy can depend on known immune response gene polymorphisms (48). Moreover, adjuvants appear to influence the V gene usage in B cells during recall responses (49), improve the breadth and affinity of antibodies after vaccination (50) and affect the extent of somatic hypermutations (51). The mechanisms are unknown. However, a study in mice by Malherbe et al. found that monophosphoryl lipid A (MPL) administration resulted in a higher affinity within the TCR repertoire, compared to alum and complete Freund's Adjuvant (52). Taken together, personalized formulations provide opportunities to improve the magnitude and breadth of vaccine responses to qualitatively and quantitatively improve immune memory and possibly reduce side-effects (53).

Personalization of vaccines can be achieved in several ways. Vaccines can be tailored for groups of individuals defined by common characteristics such as age, sex, ethnicity, HLA-type, Fc-gamma-receptor polymorphism etc., or they could be personalized for each individual to optimally engage the immune repertoire. However, immune repertoire-tailored vaccines might not even have to be personalized to the individual level. For example, population group-specific differences in T cell repertoires have been described in flu vaccination trials when comparing adults and elderly responses. These studies showed that the elderly (>60 years old) showed a reduced proportion of a public clonotype (TRAV27-TRBV19) and an increased proportion of private clonotypes amongst flu-specific T cells compared to adults (25–58 years old) (54). This suggests that different immunogens might be needed to optimally generate or boost responses in different age groups. Autoimmune diseases like Type 1 diabetes can also profoundly affect the T cell repertoire (55). Vaccines therefore have to be tailored for healthy and immunocompromised individuals accordingly.

Vaccine personalisation can have different levels of complexity. A personalization approach based on population groups requires a relatively small number of vaccine formulations, whereas high individualization requires a large library of different vaccines (Figure 2A). For infectious diseases, where low cost of the vaccine is often critical, only the former approach is currently feasible. The main argument has been that individualized vaccines are not necessary to protect from infection and that the additional cost is not justified (53). Nevertheless, a compromise could easily be reached by making an economically feasible limited number of different vaccine formulations in order to provide effective options for individuals with different immune status (53).

To develop personalized vaccines, which take into account repertoire information, measurable sets of criteria determining BCR/TCR responses to antigen must be established first. So far, the scientific field is determining pathogen-specific sequence motifs after immunization, i.e., retrospectively (Tables 1, 2). Overall, only a small number of pathogens have been studied and the associated epitopes are usually highly immunogenic, generating relatively easily traceable clones of expanded B or T cells. This means that more and larger databases must be established before the field can move to predicting vaccine responsiveness (Figure 2B).

In keeping with this, the same CDR3 sequences can be assembled from different V and J alleles, with evidence from human influenza-specific memory B cells (21), HIV-specific antibodies (14) and from EBV, CMV, and influenza-specific CD8 T cells (34). This means that a well-designed antigen could engage BCRs assembled from different germline alleles, which after somatic hypermutation can converge into the same or very similar CDR3 sequence and reach a high level of protection. Theoretically, an antigen that binds to more abundantly expressed V and J alleles could be more successful in this scenario. Separately, the same TCR or BCR might also bind to several antigens. Showing this concept of promiscuity for BCR binding, Manivel et al. tested the binding of mouse germline antibodies to a large random 12-mer peptide phage display library and demonstrated that each antibody bound several peptides and that peptides could also be recognized by different antibodies (56). These examples illustrate how complex it will be to identify the best fitting vaccine for a given repertoire. However, given that best fit predictions are a physicochemical and mathematical problem, machine learning and artificial intelligence will probably provide solutions in the near future.

## Using Single Cell RNAseq Technology to Sequence Lymphocyte Receptors and Assess the Transcriptome

How can single cell RNA sequencing assist in the development of personalized vaccines?

Commercial kits/protocols and services to analyze both the transcriptome and the TCR or BCR variable region of single cells are now available (10xGenomics, iRepertoire, and others). Combined total mRNA- and VDJ sequencing provide insight into important transcriptional heterogeneity of antigen-specific vs. non-specific lymphocytes and clonal relationships of cells in different subsets. Technically, 5′ template-switching methods such as Smart-seq2 (57) that cover the full length mRNA sequence are necessary since sequencing 50–150 bases starting from the 3′ end will miss the essential part, namely the rearranged VDJ region of immune receptor that is unique for each cell or expanded clone (Figure 1). 10xGenomics offers a 5′ targeting protocol for VDJ sequencing and total mRNA sequencing. The beads in the conventional transcriptome sequencing protocol capture the 3′ end of the mRNA.

Whole transcriptome analysis of single cell (scRNAseq) can identify new B and T cell subsets, which may not be obvious based on conventional surface marker stains, or in cases where populations only differ based on mRNA expression but not based on protein expression. Importantly, the possibility to clone and recombinantly express BCRs and TCRs from single cells of interest enables functional validation. This can simply mean verification of binding, but monoclonal antibodies or TCR-transfected cells also enable the analysis of neutralization for mAbs, or the detailed TCR-MHC binding mechanism for T cells. Such detailed analysis can confirm the biological role of new B or T cell subsets. Independently, the establishment of more robust techniques to sort antigen-specific B cells has facilitated assessment of correlations between transcriptome, phenotype, variable region sequence and antigen-specificity (58).

Analysis of transcriptome beyond the established rearrangement of TCR/BCR is relatively new and there are few publications (59–63). However, this is expected to increase as a result of 10xGenomic's launch of the 5′-VDJ single cell sequencing kits in 2017. The power of single cell technology is 2-fold: (1). it provides the opportunity to validate individual BCRs and TCRs and (2). It provides transcriptome information of the cell expressing the BCR or TCR. Although there are few studies yet to show a correlation of cell phenotype with antigen-specificity this remains an exciting area for future studies (64, 65) and could provide additional avenues for vaccine monitoring and companion diagnostics.

## Conclusions

The identification of converging sequences is complicated by their low frequency and by the constant turnover of B and T cell repertoires. The studies summarized in Tables 1, 2 each describe only one or few converging sequences or sequence motifs. Therefore, an obvious limitation for predicting immune responses and designing effective vaccines is the volume of information on convergence. Huge TCR and BCR libraries for targeted infections must be available for machine learning to be effective.

It is probable that the number of identified public disease-specific clonotypes will increase exponentially with developing technology that has a higher throughput and is cheaper. For example, the Human Vaccine Project and notably its Human Immunome arm (https://www.humanvaccinesproject.org/work/human-immunome-program/) work toward a comprehensive database of the human immune repertoire. Such massive consolidated efforts will over time result in databases that will eventually become useful for clinical application.

Attempts to standardize sequence data formats and develop curated databases are ongoing, for example by the AIRR group (http://airr.irmacs.sfu.ca/home). To leverage the expertise of the community, it will be essential that databases are publicly available, well-maintained and continuously updated.

Box 1 provides a list of recent technical and practical advances that will facilitate the discovery and validation of potential specific sequence motifs.

Box 1 **Recent technical and practical advances for sequence-function analysis:**

– Large and curated databases, such as VDJdb (66, 67) and McPAS-TCR (67)
– Algorithms that can identify shared CDR3 motifs and that are able to pick up similar motifs from CDR3 sequences that are not necessarily very similar on the nucleotide level (4)
– Algorithms that take into account structural information of immune receptor-epitope interaction (4, 39, 40, 68)
– Algorithms that can predict specific convergent sequences based on small number of subjects with the same infection/vaccination (26)
– Validation of specificity on single cell/molecular level by recombinant expression of mAbs or TCRs

A limitation of previous studies is the focus on highly immunogenic epitopes that are normally present in relatively high frequencies (Tables 1, 2). Even these high-frequency sequences induced in the context of persisting viral infections or highly immunogenic challenges such as a Yellow Fever vaccination are not trivial to find. Identifying much rarer clones is therefore expected to be more challenging, even at bulk level. There is limited opportunity to identify specific effective clones with high-throughput single cell sorting technologies. A combination of bulk and single cell analysis will therefore remain relevant.

An important proof of principle for specific converging sequences has been demonstrated for antigens that are highly immunogenic and generate high frequencies of a given TCR or BCR. Going forward, more focus must be on new pathogens. For example, interesting candidates could be those causing common infections that affect large populations, enabling the discovery of common footprints in immune repertoires. Such studies will eventually enable analysis of the historical immune response to infections based on the TCR/BCR repertoire.

The history of antigen exposure is a factor that drives immune repertoire diversity in each individual. Females appear to develop a more diverse T cell repertoire compared to males, with the largest difference observed during adulthood (30–50 years) (69). In the context of vaccination, immunizations of newborns reduces the immune repertoire variability that is driven by exposure. Different vaccine formulations could be designed based on naïve repertoires in newborns or possibly based on gender in adults.

In summary, research on the immune repertoire is still in its infancy with regard to clinical application for infectious disease vaccination, diagnosis, and prognosis. However, the efforts in establishing large repertoire databases and the availability of single cell technology will facilitate progress in understanding how the immune repertoire will improve biomedical applications.

## Author Contributions

The author confirms being the sole contributor of this work and has approved it for publication.

### Conflict of Interest Statement

The author declares that the research was conducted in the absence of any commercial or financial relationships that could be construed as a potential conflict of interest.
